# Talking therapies for anxiety and depression in people affected by dementia: A scoping meta‐review

**DOI:** 10.1111/papt.70003

**Published:** 2025-07-31

**Authors:** Georgina Charlesworth, Cassie Eastham, Lydia Morris

**Affiliations:** ^1^ Research Department of Clinical, Educational and Heath Psychology University College London London UK; ^2^ North East London NHS Foundation Trust London UK; ^3^ Greater Manchester Mental Health NHS Foundation Trust Manchester UK; ^4^ Division of Psychology & Mental Health the University of Manchester, School of Health Sciences University of Manchester Manchester UK

**Keywords:** caregiver, carer, CBT, coping, meta‐analysis, mindfulness, review

## Abstract

**Background:**

Symptoms of anxiety and depression are common in people affected by dementia (people with dementia and family carers). There are many reviews of psychological interventions to reduce stress and distress in people with dementia and family carers, but little cross‐fertilisation with the evidence base underpinning NHS Talking Therapies for anxiety and depression (TTad).

**Objectives:**

To review the psychological intervention literature for people with dementia and their family carers through the lens of TTad, thus providing a bridge between the two literatures.

**Methods:**

We undertook a rapid scoping review of meta‐analyses of TTad‐recommended therapies used with people with dementia and family carers, including cognitive‐behavioural therapies (CBT), behavioural activation, problem‐solving, counselling for depression and mindfulness‐based cognitive therapy.

**Results:**

All meta‐analytic reviews of cognitive and behavioural therapies (nine with family carers and a Cochrane review with people with dementia) demonstrated the effectiveness of behavioural activation and CBT‐informed interventions in reducing symptoms of depression. Anxiety was less frequently studied, with no positive findings found for people with dementia. Psychoeducation was the most effective approach for anxiety reduction in carers. Evidence from meta‐analyses of mindfulness‐based interventions (MBIs) for carers showed reduced symptoms of both depression and anxiety.

**Conclusions:**

There are ontological and methodological differences in the literatures underpinning TTad and psychological interventions for people affected by dementia. To improve access to TTad for people with dementia and family carers, changes are required to research methodologies, TTad data collection and approaches to service delivery.

**Practitioner Points**

- CBT‐informed interventions can reduce depression in people with dementia and family carers, including online interventions for carers
- Psychoeducation is effective in anxiety reduction, plus mindfulness‐based interventions for carers but not people with dementia
- Positive practice guidance for Talking Therapies for anxiety and depression (TTad), now within the TTad manual (v.7), includes recommendations for flexibility and adjustment

## INTRODUCTION

In the UK, there are an estimated 850,000 people with dementia, with 676,000 in England (www.england.nhs.uk/mental‐health/dementia/). The majority of people with dementia (also termed ‘major neurocognitive disorder’; DSM‐V, 2013) are supported by one or more family members or close friends. Receiving a diagnosis of dementia can have a significant psychological impact for both the individual and their support network, especially for those with young‐onset diagnoses (before 65 years of age). People with dementia and their family carers are at elevated risk of depression (NHS England, 2024), and symptoms of anxiety and depression are common (Leung et al., 2021).

In England and Wales, commissioning for dementia services currently focusses on meeting national diagnostic targets. Regarding post‐diagnostic support, the Department of Health and Social Care's guide for people with dementia makes no reference to psychological therapies (Department of Health and Social Care, 2018), although NICE guidance for dementia (NG97; National Institute for Health and Care Excellence, 2018) recommends group psychoeducation for carers and psychological treatments people with mild to moderate dementia who have mild to moderate depression and/or anxiety. The ‘looking after someone’ section of the ‘living with dementia’ pages of the NHS website provides a direct link to ‘find a Talking Therapies Service’ if family carers are ‘struggling to cope’, ‘finding it difficult to talk about the stress involved with caring’ or feeling like they are ‘not managing’ (National Health Service, n.d.).

Talking Therapies for anxiety and depression (TTad) services [formerly known as ‘Improving Access to Psychological Therapies’ or ‘IAPT’ services] provide treatment for adults with mild to moderate depression and anxiety disorders, and (since 2018) for people who have long‐term conditions (LTC) or other persistent physical symptoms in the context of depression and anxiety disorders (NHS England, 2018). The therapies offered by TTad are consistent with NICE recommendations for treatment of mild to moderate anxiety and depression (NHS England, 2024, pp. 17–19; NHS Futures, 2024). Three categories of depression (‘less severe’, ‘more severe’ and ‘chronic’) are considered separately to eight anxiety diagnoses. Services are organised into ‘steps’. Low‐intensity (LI) interventions (‘step 2’; typically up to six, 20‐ to 30‐minute sessions) include individual guided self‐help based on cognitive behaviour therapy (CBT) principles, behavioural activation (BA), problem‐solving therapy (PST) and psychoeducation (written or electronic). ‘Step 3’, high‐intensity (HI) treatments include individual CBT, person‐centred experiential counselling for depression (PCECfD), brief Psychodynamic Therapy and Interpersonal Psychotherapy (IPT). With the exception of group exercise, which is recommended for all levels of depression, group delivery is only recommended for less severe depression (group CBT, group BA and group Mindfulness‐Based Cognitive Therapy (MBCT)). For anxiety, specific HI CBT manuals are recommended for each diagnostic category, some with and some without LI options. The target outcome is for the service user to ‘reliably recover’, ending treatment below the threshold for ‘caseness’ on self‐report measures of depression (PHQ‐9) and anxiety (GAD‐7). Where service users do not show post‐LI recovery, or if LI interventions are not recommended for a particular diagnosis, service users can be ‘stepped up’ to receive ‘high‐intensity’ (HI) interventions from accredited practitioners (NHS England, 2024).

The extent to which family carers of people with dementia engage with, and benefit from, psychological interventions in TTad services is not known due to carer status not being recorded in the TTad minimum dataset. By contrast, there is practice‐based evidence of outcomes for people with dementia in TTad, albeit for very small numbers. Between 2012 and 2019, 1549 people with dementia identified as ‘cases’ for anxiety or depression (0.08% of the total population of IAPT users; Bell et al., 2022). Depression and anxiety scores showed significant improvement over the course of therapy (defined as two or more sessions; mean number of TTad sessions 5.53 (SD 3.98)). Reliable improvement was seen in 62% and reliable recovery in 40%, although propensity‐matched controls without dementia showed greater improvements, and 9% of people with dementia reliably declined (Bell et al., 2022).

Separate from the national organisation of TTad, there is a long‐standing and extensive international research literature on non‐pharmacological interventions for family carers of people with dementia, as illustrated by multiple ‘umbrella’ or meta‐reviews (Cheng & Zhang, 2020; Gilhooly et al., 2016; Jütten et al., 2018), including reviews on ehealth and web‐based interventions (Naunton Morgan et al., 2022). The wide range of demands on carers, and heterogeneity in characteristics and circumstances (e.g. kinship and quality of relationship, cohabitation or otherwise, type and stage of dementia in care‐recipient, secondary stressors) mean that a multitude of interventions have been developed, with an associated challenge for intervention classification. Many interventions in the research literature are multi‐component, including psychoeducation about dementia, skills‐based training and tangible support. For people with a dementia diagnosis, the literature on psychological interventions has been focussed on improving neurocognition and quality of life. However, over the past decade or so, the evidence base for psychological therapies for mood disorders has started to grow (Sukhawathanakul et al., 2021).

At present, there is no meaningful cross‐referencing between NICE guidelines for depression or specific anxiety diagnoses (NHS England, 2024) and either NICE guidelines for dementia (NG97; National Institute for Health and Care Excellence, 2018) or adult carers (NG150; National Institute for Health and Care Excellence, 2020). TTad Positive Practice Guidance (PPG) for working with older people (BABCP, 2024) has been incorporated into the TTad manual, (v. 7; NHS England, 2024) and includes recommendations to increase engagement with people with dementia and family carers. However, the only specified protocol (‘Strategies for Relatives’, Livingston et al., 2013, 2020) is an 8‐hour, face‐to‐face, individually‐delivered intervention, thus being incompatible with the ‘hard stop’ of six 20‐min sessions for LI CBT in many Step 2 services. The aim of this paper is therefore to review the literature on psychological interventions for anxiety and depression in people with dementia and family carers through the lens of TTad Manual (NHS England, 2024). By doing so, we hope to improve access to appropriate and effective interventions for anxiety and depression in people with dementia and family carers living in the community.

## METHOD

We undertook a rapid scoping review by (1) inspecting the TTad Manual (v.7; NHS England, 2024) and NHS Talking Therapies Treatment Packages Resource (NHS Futures, 2024) to identify recommended psychological therapies for anxiety and depression, (2) searching Google Scholar using the names of TTad‐recommended therapies as search terms alongside the term ‘meta‐analysis’ and synonyms for ‘dementia’ (cognitive impairment, Alzheimer's) plus ‘caregiving’ (caring, carer), (3) reading search outputs and reference lists of umbrella and meta‐reviews to identify meta‐analyses of randomised controlled trials (RCTs) of psychological therapies for anxiety or depression in community‐dwelling people with dementia or their family carers, (4) undertaking forwards and backwards citation checking of relevant meta‐analyses to identify additional relevant meta‐analyses.

We included reviews with meta‐analyses of the effectiveness of TTad‐recommended interventions on measures of anxiety and depression in populations of people with dementia and their family carers. We had no restriction on comparator (control) interventions. We included both LI and HI forms of intervention and all modes of delivery (individual and group; people with dementia and family carers separately or together; face‐to‐face, telephone, video conference, workbooks). We included reviews of mindfulness‐based interventions (MBIs) that included MBCT (a TTad‐recommended therapy) and reviews that included people with mild cognitive impairment, as long as studies on people with dementia were also present. We excluded reviews where the meta‐analysis was within‐intervention only, with no consideration of control conditions, or where the focus was exclusively on people living in long‐term care settings.

For eligible meta‐analyses, we extracted the following data for tabulation: lead author, year, number of included studies, eligibility criteria (PICOS: population, intervention, comparator/control, outcomes of interest; study design), interventions used and outcome effects as identified through meta‐analyses.

## RESULTS

Of 22 relevant meta‐analyses identified, five were excluded due to meta‐analyses using intervention only (pre‐post) meta‐analyses without comparison to control (Collins & Kishita, 2019; Collyer & Dorstyn, 2024; Molero Jurado et al., 2020; Robinson & Moghaddam, 2022; Verreault et al., 2021).

### Characteristics of included reviews

Of the 17 included meta‐analytic reviews, four focussed on outcomes for people with dementia (Table 1), including one Cochrane review (Orgeta et al., 2022), and 13 focussed on outcomes for carers of people with dementia (7 CBT‐based, 4 MBI, one BA only and one including CBT, MBI and BA meta‐analyses; Table 2) which included two Cochrane reviews (Liu et al., 2018; Vernooij‐Dassen et al., 2011). The included reviews were published between 2011 and 2024, with the most recent searches being in June 2023 (Saragih et al., 2024). The number of trials in the reviews ranged from eight to 29 for people with dementia and five to 140 for carers.

**TABLE 1:** Meta‐analyses of TTad‐recommended interventions for people with dementia (chronological).

Author; number of studies and year	Eligibility criteria	Interventions	Findings
Wang et al. (2020) 9 studies Database inception to June 2019	*Participants*: any type of dementia; subjective cognitive complaints, MCI, early dementia *Interventions*: MBSR, MBCT, other combinations of mindfulness and behavioural therapies or meditation‐based interventions. Excluded: ACT, DBT *Comparators*: not specified *Outcomes*: depression, anxiety, stress, QoL *Study design*: RCT, controlled, pre‐post	Interventions: MBSR (2 studies); mindfulness‐based interventions (5 studies); meditation (2 study)	*Depression*: Favours MBI SMD = −0.39 (95% CI −0.62 to −0.15) *Anxiety*: ns *Stress*: ns *Quality of Life*: ns
Nagaoka et al. (2021) 8 studies Database inception to Jan 2020	*Participants*: older adults with MCI or dementia (excl. DLB, FTD, HD, PD) *Interventions*: >4 × 1 h. mindfulness meditation (Exclusions: other meditation interventions and mindfulness without meditation, e.g. ACT) *Comparators*: Active control, waitlists, TAU *Outcomes*: Anxiety, depressive symptoms, cognitive functions, QoL, mindfulness, ADL *Study design*: RCTs	Interventions: MBSR (3 studies); mindfulness‐based interventions (4 studies); mindfulness program (1 study) Controls: psychoeducation, cognitive games, usual care, waitlist	*Depression*: ns *Anxiety*: ns *Cognitive function*: ns post‐intervention; favours MBI medium‐ to long‐term SMD = 1.19 (95% CI 0.68 to 1.71) *Quality of Life*: ns *Mindfulness*: favours control post‐intervention SMD = −1.20 (95% CI −1.84 to −0.56) and medium‐ to long‐term SMD = −1.29 (95% CI −1.94 to 0.65) *ADL*: favours MBI medium‐ to long‐term SMD = −1.08 (95% CI −1.60 to −0.57)
Orgeta et al. (2022) 29 (25 included in meta‐analyses) Database inception to July 2019	*Participants*: dementia or MCI *Interventions*: any based on psychological theory and designed to reduce anxiety and depression, improve adaptive functioning or both *Control*: usual care or comparison group receiving no specific psychological intervention *Outcomes*: depression, anxiety, QoL, cognition, ADL, neuropsychiatric symptoms *Study design*: RCTs, inc cluster‐randomised	Supportive and counselling therapies (11 studies) BA (8 studies) CBT (4 studies) Problem‐solving therapy (3 studies) Mindfulness‐based interventions (3 studies) Interpersonal therapy (1 study)	CBT (including BA+PST) *Depression*: favours intervention SMD −0.23 (95% CI −0.37 to −0.10) *QoL*: SMD −0.31 (95% CI −0.13 to −0.50) *ADL*: 0.25 (95% CI −0.4 to −0.09) ns: Anxiety, neurocognition Supporting and counselling interventions: ns – all outcomes MBI: not enough data for meta‐analysis
Han (2022) 11 articles (10 RCTs) Database inception to March 2021	*Participants*: older adults with MCI or dementia *Interventions*: MBSR; MBCT; other variations facilitating mindfulness; exclusions: multi‐component of which mindfulness only a small part; exercise and physical activity interventions *Outcomes*: Psychological symptoms (e.g. depression), cognitive functions (e.g. memory), quality of life *Study design*: RCT	MBSR (6 studies); MBSR/MBCT combination (2 studies); other various (2 studies) All interventions delivered as in‐person groups Controls: both active (e.g. health education; neurocognitive rehab) and passive (usual care)	*Depression*: favours control post‐intervention SMD = −0.3 (95% CI −0.56 to −0.04) and follow‐up SMD = −1.32 (95% CI −2.39 to −0.25) *Anxiety*: ns post‐intervention; favours control at follow‐up SMD = −2.16 (95% CI −3.61 to 0.70) *Memory (delayed recall)*: favours control post‐intervention SMD = −0.26 (95% CI −0.52, −0.0); ns at follow‐up *QoL*: favours control: SMD = 0.81 (95% CI 0.04, 1.57)

Abbreviations: ACT, acceptance and commitment therapy; ADL, activities of daily living; BA, behavioural activation; DBT, dialectical behaviour therapy; DLB, dementia with lewy bodies; FTD, frontotemporal dementia; HD, Huntington's disease; MBCT, mindfulness‐based cognitive therapy; MBI, mindfulness‐based intervention; MBSR, mindfulness‐based stress reduction; MCI, mild cognitive impairment; PD, Parkinson's disease; QoL, Quality of Life; RCT, randomised controlled trial; TAU, treatment as usual.

**TABLE 2:** Meta‐analyses of TTad‐recommended interventions evaluated with family carers of people with dementia/neurocognitive disorders.

Author	Eligibility criteria	Interventions	Findings
Cheng et al. (2020) 140 articles representing 131 programmes Search dates: NR	*Population*: informal carers of people with dementia *Interventions*: MBIs (inc MBCT), psychoeducation, counselling and psychotherapy (inc CBT, BA), support groups, multi‐component *Comparator*: not specified *Outcomes*: burden/stress, depressive symptoms, anxiety, subjective wellbeing, physical health, social support, positive aspects of caring, ability/knowledge (including coping, self‐efficacy and knowledge) *Study design*: RCT (incl. cluster)	MBIs (inc MBCT) Pychoeducation Counselling and psychotherapy (inc CBT, BA) Support groups Dyads Multi‐component	MBIs *Depression*: Hedge's g = −0.35 (95% CI −0.55 to −0.15) *Anxiety*: Hedges' g = −0.25 (95% CI −0.47 to −0.03) ns—burden/stress, subjective wellbeing, ability/knowledge
Hopkinson et al. (2019) 25 studies Search dates: 1996–2016	*Population*: Primary informal carers of people with dementia with clinically significant psychological morbidity (e.g. depression, anxiety) at baseline on psychometric tools (no diagnosis required) *Intervention*: CBT or ‘CBT principles’‐based (i.e. sought to achieve change in participants' thoughts, emotions and/or behaviours) delivered in person or via telephone by trained professionals. Excl: Multi‐component interventions with >25% non‐CBT content; > 50% content ‘third‐wave’ CBT; web‐based, prerecorded content or real‐time video. *Comparator*: waiting list; usual care; alternative psychotherapeutic intervention *Outcomes*: *d*epression, anxiety, stress *Study design*: *RCTs* and controlled trials	Group CBT‐informed, e.g. ‘Coping with Caregiving’; resourcefulness training Individual CBT‐informed, e.g. ‘Strategies for relatives’ Dyadic counselling group, e.g. Systems and cognitive‐behavioural approach to assist problems identification, stress reduction, frustration management, communication and conflict resolution	*Depression*: favours CBT: SMD = −0.34 (95% CI −0.47 to −0.21) Subgroup analyses: group interventions associated with significant reductions, but not individual; no difference between interventions using 8+ sessions vs. <8 *Anxiety*: *ns* *Stress*: favours CBT: SMD = −0.36 (95% CI –0.52 to 0.20)
Kaddour et al. (2019) 12 publications Search dates: Database inception to July 2017	*Population*: adult informal carers of a person with dementia. *Intervention*: based on CBT theory or techniques. ‘Low intensity’ (defined as: interventions delivered by facilitators locally licensed/accredited to practice CBT independently, lasting no more than 6 hours; interventions delivered by facilitators who are not licensed/accredited to practice CBT independently, lasting any duration; and self‐help interventions, p. 963) *Comparator*: waitlist, treatment as usual, placebo or non‐active intervention. *Outcomes*: anxiety, depression, burden, distress *Study design*: RCT or controlled clinical trial. Exclusions: studies rated as ‘very poor’ or ‘exceptionally poor’ using RCT Psychotherapy Quality Rating Scale (RCT PQRS)	Multi‐component CBT‐based, with or without non‐CBT components, e.g. education on local resources Group CBT (5 studies), e.g. ‘Coping with Caregiving’ course Individual CBT‐informed (9 studies) Self‐help, e.g. iCare Stress Management e‐training Videoconference, telephone and web‐based delivery	*Depression*: favours CBT. Hedge's g 0.27 (95% CI 0.15 to 0.39) *Anxiety*: favours CBT. Hedge's g 0.58 (95% CI 0.19 to 0.97) *Burden*: favours CBT (Hedges g 0.53; 95% CI 0.16 to 0.90) *Stress/Distress*: favours CBT Hedges g 0.33 (95% CI 0.17 to 0.48)
Kishita et al. (2018) 31 studies Search dates: to Nov 2016	*Population*: dementia family carers *Intervention*: non‐specified for search; post‐search categorisation for: case management, provision of information on dementia and care‐related issues, communication training, CBT techniques for managing carers' emotional difficulties, non‐CBT techniques for managing difficulties related to caregiving, behaviour modification *Comparator*: non‐active control *Outcomes*: depression, anxiety or burden as primary outcome *Study design*: randomised	(i) CBT‐based psychotherapeutic interventions cognitive and behavioural strategies to address specific misconceptions or maladaptive assumptions OR acceptance of distressing thoughts and emotions (10 face‐to‐face; 5 technology mediated) (ii) Psychoeducation skills building increasing carers' knowledge of dementia and teaching specific coping skills for managing common emotional distress and/or behavioural changes associated with dementia (12 face‐to‐face, 4 technology mediated)	CBT‐based *Depression*: Hedge's g = 0.53 (95% CI 0.22 to 0.84) *Anxiety*: Hedge's g = 0.84 (95% CI = 0.27 to 1.41) *Burden*: ns Psychoeducational skills building *Depression*: ns *Anxiety*: no data reported *Burden*: Hedge's g = 0.18 (95% CI = 0.06 to 0.29)
Kor et al. (2018) 5 studies (3 in meta‐analysis) Search dates: 1990–2016	*Population*: family carers aged >18 years of people diagnosed with dementia. Other chronic diseases included if >75% of cases had dementia diagnosis *Intervention*: mindfulness as major component *Comparator*: usual care or active controls *Outcomes*: stress, depression, anxiety, burden *Study design*: RCTs and quasi‐experimental studies	MBSR (4) MBCT plus dementia education (1)	*Depression*: favours intervention SMD = −0.62 (95% CI −0.97 to −0.27) *Stress*: favours intervention SMD = 0.57 (95% CI 0.23 to 0.92) Anxiety: no data *Burden*: ns
Kwon et al. (2017) 12 studies Search dates: Database inception to Nov 2016	*Population*: carers of people with dementia *Intervention*: CBT *Control*: untreated or minimally treated (other therapeutic modalities excluded) *Outcomes*: depression, perceived stress, memory and behavioural problems, self‐efficacy, burden, dysfunctional thoughts about caregiving, health or symptoms, coping and anxiety *Study design*: *RCTs*	CBT‐based content inc coping skills, thought modification, building social support and engagement in pleasant activities Delivered individually or in groups via telephone web, or face‐to‐face	*Depression*: favours CBT. Pooled mean difference − 5.02 (−7.52 to −2.52) Narrative synthesis only for other outcomes
Liu et al. (2018) 5 studies Search dates: Database inception to Sept 2017	*Population*: informal carers of people with dementia *Interventions*: MSBR with or without adaptations *Comparator*: active‐control interventions, waiting list or usual care *Outcomes*: depressive symptoms, anxiety, burden, coping style, quality of life, dropout rates *Study design*: Individually randomised, parallel‐group controlled trials. Ecl.cluster‐randomised and cross‐over	MBSR (4 studies) Mindfulness meditation (1 study) Active controls: education, social support, progressive muscle relaxation Inactive controls: self‐help education; respite care	*MBSR* vs *active control* *Depression*: favours intervention SMD = −0.63 (95% CI −0.98 to −0.28) *Anxiety*: favours intervention. MD −7.5; 95% (CI −13.11 to −1.89) *Burden*: ns *MBSR* vs *inactive control*: ns for all outcomes No data for coping style or QoL
Saragih et al. (2024) 13 studies Search dates: Database inception to June 2023	*Population*: dementia caregivers *Interventions*: any therapy related to mindfulness *Comparator*: unspecified *Outcomes*: depression, anxiety, stress, burden *Study design*: RCTs	MBCT (4 studies) Yoga (3 studies) Meditation (3 studies) Control: telephone monitoring, no treatment or ‘normal daily activities’	*Depression*: ns overall and <8 sessions. For 8+ sessions favours intervention SMD = −0.38 (95% CI −0.73 to −0.03) *Anxiety*: favours intervention SMD = −0.58 (95% CI −1.10 to −0.05) *Stress*: favours intervention SMD = −0.49 (95% CI = −0.80 to −0.18) *Burden*: ns
Scott et al. (2016) 4 studies Search dates: not reported	*Population*: primary informal carers of care‐recipient with dementia living in community *Intervention*: ‘pure’ technology‐based self‐help CBT delivered by flexible format (internet; DVD; video) with at least one cognitive and one behavioural component *Comparat*or: unspecified *Outcome*: carer psychological functioning or wellbeing *Study design*: RCTs and quasi‐experimental	Psychoeducational component and at least one other aspect of cognitive or behavioural therapy (e.g. cognitive restructuring, behaviour modification)	*Depression*: favoured intervention SMD = −0.27, 95% CI: −0.02 to −0.52
Sun, Ji, Leng, Li, et al. (2022) 85 studies Search: inception to March 2021	*Population*: informal carers of people with dementia living in community *Interventions*: psychoeducation, counselling, CBT, BA, ACT, MBI, case management, support group, music, exercise, multi‐component *Comparator*: any other type of non‐pharmacological intervention, or brief intervention (e.g. leaflet), or no‐treatment control (waitlist, TAU) *Outcomes*: depression, anxiety, QoL, carer burden *Study design*: RCTs only	*TTad compatible*: CBT (14 studies); MBI (11 studies); BA (5 studies) *Other interventions*: psychoeducation (31 studies); multi‐component (17 studies); support (11 studies); counselling (6 studies); case management (5 studies); exercise (3 studies), music (1 study), ACT (1 study)	*Depression*: BA vs. control: BA favoured: SMD = −1.37 (95% CI −1.90 to −0.85). MBI vs. control: MBI favoured SMD = −0.78 (95% CI −1.16 to −0.41). CBT vs. control: CBT favoured SMD = −0.53 (95% CI −0.92 to −0.14) *Anxiety*: psychoeducation favoured: SMD = −0.53 (95% CI −0.97 to −0.09). ns—CBT, MBI, multi‐component, case management, support groups *Burden*: psychoeducation favoured: −0.60 (95% CI −0.87 to −0.33)
Sun, Ji, Leng, & Wang (2022) 37 studies Search dates: Database inception to Jan 2022	*Population*: informal caregivers currently providing caregiving support to people with dementia *Intervention*: any structured and conceptualised CBT (individual or group (face‐to‐face), telephone, internet, combined delivery formats) *Comparators*: any other types of CBT formats, no treatment, treatment, treatment as usual or brief information‐based support, e.g. leaflet *Outcomes*: depressive symptoms *Study design*: *RCTs*	Individual (6 studies) Group (12 studies) Internet (6 studies) Telephone (14 studies) Combined (3 studies) Intervention durations: 5 weeks to 12 months	*Depression*: CBT intervention favoured over control for delivery by: Internet: SMD = −1.33 95% CI (−2.18 to −0.66) Telephone: SMD = −1.29 (95% CI −1.89 to −0.61) Individual face‐to‐face: SMD −1.04 (95% CI –2.01 to −0.07) Group face‐to‐face: ns Combined: ns
Vernooij‐Dassen et al. (2011) 11 studies Search: database inception to Apr 2009	*Population*: family carers of a person with dementia living in community. *Intervention*: ‘cognitive reframing’ (inc interventions based on stress coping, stress management and CBT) *Control*: usual care *Outcomes*: psychological morbidity (inc. depression, anxiety), quality of life, self‐appraisal of role performance (inc burden, coping and self‐efficacy) and health care utilisation of the person with dementia (e.g. admission to residential care) *Study design*: RCTs	CBT (7 studies) inc psychoeducation, skills training and practice, modification of ‘dysfunctional thoughts’. E.g. (Márquez‐González et al., 2007); Marriott et al. (2000) cognitive‐behavioural family intervention Stress coping (4 studies) inc web‐based, multimedia; video‐assisted modelling; skills building and psychoeducation Stress management (1 study) inc family counselling and support	*Depression*: favours intervention. SMD −0.66 (95% CI –1.27 to −0.05) *Anxiety*: favours intervention. SMD −0.66 (95% CI –1.27 to −0.05) *(Dis)stress*: intervention favoured. SMD −0.24 [95% CI −4 to −0.07] ns—coping, self‐efficacy, burden, reaction to the person with dementia's behaviour, institutionalisation of person with dementia
Xu et al. (2020) 10 studies Search dates: Mar 1988 to Sept 2019	*Population*: family caregivers of people with dementia *Intervention*: based on BA theories *Control*: not specified *Outcomes*: not specified *Study design*: RCT	Interventions based on Lejuez and Lewinsohn. Also cognitive‐behavioural components, e.g. analyses of barriers to engaging in pleasurable activities Comparator: usual care; information packages; psychoeducation	*Depression*: intervention favoured over control. SMD –0.69; 95% CI –1.12 to −0.25 Other outcomes (anxiety, hostility, frustration, strain) described narratively only

*Note*: One intervention designated ‘MBCT’ by Saragih et al. (2024) was designated ‘mindfulness meditation’ by Liu et al. (2018) and ‘MBCT plus dementia education’ by Kor et al. (2018). Two interventions designated ‘yoga’ in Saragih et al. (2024) were classified as MBSR by Liu et al. (2018) and Kor et al. (2018).

Abbreviations: BA, behavioural activation; CBT, cognitive behaviour therapy; CI, confidence interval; MBCT, mindfulness‐based cognitive therapy; MBSR, mindfulness‐based stress reduction; ns, not significant; QoL, quality of Life; RCT, randomised controlled trial; SMD, standardised mean difference.

Although we restricted inclusion to reviews of TTad‐recommended interventions used with people with dementia or family carers and anxiety or depression as outcomes, there was still considerable variability between review eligibility for populations, interventions, comparators and outcomes:

### Populations

All reviews for people with a dementia diagnosis also allowed inclusion of people with mild cognitive impairment (MCI). Most considered dementia in general, with one excluding specific dementia subtypes (Nagaoka et al., 2021). Two reviews specifically required that informal (family) carers must be caring for persons with dementia living in the community (Sun, Ji, Leng, Li, et al., 2022; Vernooij‐Dassen et al., 2011), but none of the reviews were restricted to a specific kinship, stage or duration of caregiving. One review restricted eligibility to trials where carers had clinically significant psychological morbidity identified by psychometric tools at baseline (Hopkinson et al., 2019), but no review restricted eligibility to trials where carers had a formal diagnosis of anxiety or depressive disorder. The duration of psychological morbidity was not considered in any of the reviews.

### Interventions

Reviews focussed predominantly on CBT‐informed or mindfulness‐based interventions with no reviews of TTad‐recommended protocols for counselling (PCECfD) or couples work for depression (BCT, CTD), or for specific anxiety disorders. There were no reviews for which IPT was separately meta‐analysed. For both CBT and MBI, there was a lack of consistency in definitions (Table 2). For example, whereas CBT was, for some, defined by purpose (e.g. seeking to achieve change in participants' thoughts; Hopkinson et al., 2019), for others, CBT was defined as any intervention that included a cognitive and a behavioural technique. Some reviews drew a distinction between psychoeducation and CBT (Kishita et al., 2018; Sun, Ji, Leng, Li, et al., 2022), whereas others included psychoeducation within their CBT definition (e.g. Scott et al., 2016; Vernooij‐Dassen et al., 2011). Some reviews focussed on a specific technique (e.g. cognitive reframing; Vernooij‐Dassen et al., 2011), intervention (e.g. Behavioural Activation; Xu et al., 2020), delivery mode (e.g. technology‐mediated CBT; Scott et al., 2016) or delivery intensity (e.g. low‐intensity; Kaddour et al., 2019). Some reviews clustered (e.g. Orgeta et al., 2022 grouping trials of CBT, BA and PST for analysis), whereas others separated (e.g. comparing CBT delivered through individual, group, internet or telephone modalities; Sun, Ji, Leng, & Wang, 2022). There were no MBCT‐specific reviews or analyses. MBIs included MBCT, mindfulness‐based stress reduction (MBSR), mindfulness meditation and yoga. As for CBT reviews, the definitions used within each MBI review led to instances where trials were inconsistently categorised. For example, an intervention defined as MBSR in one review was categorised as yoga in another review and MBCT in a third.

### Comparators

For some reviews, comparators were unspecified. Some restricted eligibility to non‐active controls, others to usual care or active controls. For the network meta‐analytic reviews (Sun, Ji, Leng, Li, et al., 2022; Sun, Ji, Leng, & Wang, 2022), the comparator was any intervention other than that which formed the intervention of interest within any specific meta‐analysis.

### Outcomes

Through our TTad lens, we have a focus on symptoms of anxiety and depression. However, these were not necessarily the primary outcomes for the included reviews. Other common outcomes of interest included burden, stress and distress for carers, as well as neurocognitive function (e.g. memory) and activities of daily living for people with dementia. Other outcomes of interest might be considered potential mechanisms of action; for example knowledge, coping, self‐efficacy, mindfulness and social support. Data were included in meta‐analyses without consideration of the intended target of intervention and irrespective of a measure's status as a primary or secondary outcome. Therefore, the same studies often appeared in meta‐analyses for both anxiety and depression. Most reviews commented on the wider range of measures used for each assessment domain. The TTad outcomes of PHQ‐9 and GAD‐7 were notable by their absence. Outcome measures for depression and anxiety in people with dementia often included proxy measures and observational tools, either additional to, or in place of, self‐report.

### Intervention effects on depression

All reviews included at least one depression meta‐analysis. For carers, significant positive effects were consistently found for CBT‐informed and BA interventions (see Table 2). Inspecting studies within meta‐analyses of CBT‐informed interventions, the ‘Coping with Caregiving’ group intervention (Gallagher‐Thompson et al., 2008) and its derivatives were commonly included. Developed in the USA, this intervention includes psychoeducation, cognitive restructuring and pleasant events scheduling. It has been adapted for use outside the USA, and for delivery in different formats, for example one‐to‐one (StaRt; Livingston et al., 2024) and technology‐based (Gallagher‐Thompson et al., 2010; Kajiyama et al., 2013; Kelly et al., 2024). Also included in the meta‐analyses is an evaluation of a cognitive‐behavioural family intervention (Marriott et al., 2000) with a similar foundation to NICE‐recommended family interventions for psychosis for which HEE‐funded training courses are available. Some BA programmes were adapted from treatment manuals which would be readily recognised within TTad. For example, the six session Pleasant Events Program (PEP; Moore et al., 2013) was based on Lejuez's BA manual.

For people with dementia or MCI, pooled data for CBT, BA and PST showed benefit over control for symptoms of depression (Orgeta et al., 2022). The BA studies included some interventions delivered in care home settings, and two of the three PST studies used a variant called problem adaptation therapy (PATH). Devised for people with co‐occurring cognitive impairments, depression and physical disabilities, PATH is a 12‐week home‐delivered therapy integrating PST with carer participation and environmental adaptation (Kiosses et al., 2015).

Four of five analyses of MBIs for carers favoured intervention over control (Cheng et al., 2020; Kor et al., 2018; Liu et al., 2018; Sun, Ji, Leng, Li, et al., 2022), the exception being Saragih et al. (2024). There were no direct comparisons of MBCT and MBSR; however, Kor et al. (2018) and Liu et al. (2018) both focussed on MBSR, whereas Saragih et al. (2024) included more MBCT studies. Of the four included meta‐analyses of MBIs for people with dementia or MCI, only one found the benefit of intervention. For the other three reviews, depression outcomes were: not reported, non‐significant and favouring control.

### Intervention effects on anxiety

Only eight of the 13 carer reviews (62%) reported a meta‐analysis for anxiety. Of the five CBT meta‐analyses, three found CBT favoured over control and two were non‐significant. The included trials were similar to those in the depression meta‐analyses. MBIs were favoured over control for three of the four anxiety analyses with carers, which, combined with findings for depression, suggest that mindfulness‐based interventions, particularly MBSR, should be considered for family carers.

For people with dementia, the grouping of CBT, BA and PST interventions showed no significant benefit for anxiety outcomes, even though two studies were specifically designed to target anxiety in dementia and have published manuals. These include the ‘Peaceful Mind’ programme (Paukert et al., 2010, 2013) and a cognitive therapy based on Beck and Clark's (1997) generic cognitive model of anxiety disorders (Charlesworth et al., 2015; Spector et al., 2015). There were meta‐analyses of MBIs for anxiety, three of the four reviews focusing on people with dementia, but no significant benefits were found, irrespective of intervention or comparator.

## DISCUSSION

We sought to identify the extent to which the evidence base for psychological interventions for family carers and people with dementia maps onto therapies used in English Talking Therapies services for anxiety and depression (TTad). Through scoping meta‐analytic reviews of psychological interventions for people affected by dementia, we found that CBT‐informed and Behavioural Activation (BA) interventions can significantly reduce symptoms of depression for both people with dementia and carers, including digital delivery of CBT‐informed interventions for carers. From this, we might infer that TTad services are ideally placed to meet the psychological needs of people with dementia and family carers. However, behind the headline, we found significant differences in intervention description and delivery, and diverse approaches to outcome target and measurement.

In this discussion, we consider the challenges to bridging the two literatures, the strengths and limitations of our approach to this review and the implications of our findings for practice, policy and research.

### Challenges to bridging literatures relevant to TTad and people affected by dementia

Mapping interventions for people affected by dementia onto TTad‐recommended therapies proved difficult; there were discrepancies in intervention definition, content and classification, and in assessment and intervention for mixed anxiety and depression, chronic depression, bereavement and loss, and interpersonal difficulties.

Whilst intervention classification is a long‐standing problem (Gaugler et al., 2017), our greatest challenge was bridging the ontological differences between the diagnostic framework used in TTad and the contextual stress‐coping paradigm that informs many interventions for people with dementia and carers (Gilhooly et al., 2016; Kneebone & Martin, 2003). Similar cognitive and behavioural techniques have been used for different purposes within each framework, addressing individually situated mood states versus facilitating adaptation and adjustment to objectively challenging contexts. The longstanding use of the stress‐coping paradigm for carers likely explains why MBSR appeared so frequently in the included reviews. Despite its beneficial impact for family carers on depression and anxiety, it is not currently endorsed by TTad.

The duration of symptoms of depression was not a variable of interest in included reviews with no distinction being drawn between acute and chronic presentations. Chronic depression is characterised by continually meeting criteria for the diagnosis of a major depressive episode, or having persistent subthreshold symptoms, or having persistent low mood with or without concurrent episodes of major depression for a period of at least 2 years. Chronic depression is important to assess for several reasons: Firstly, dementia and the caring associated with it are often spread over many years; according to a large UK‐based survey, 30% of informal carers for people with dementia had been providing support for between 5 and 10 years, with 22% caring for over 10 years (Office of National Statistics, 2016–2017). Secondly, people with chronic depression may not be aware that they are depressed, and this can easily apply to family carers. Indeed, many carers would not consider themselves to have mental health needs, even when meeting diagnostic criteria for specific anxiety or depressive disorders and when managing their own LTCs or other life stressors in addition to caring. Thirdly, depression in persons with dementia may predate the dementia diagnosis, and depression is now considered a risk factor for dementia (Livingston et al., 2024). In TTad, treatments for chronic depression differ from those for recent‐onset depressive episodes (NHS England, 2024). LI interventions are not recommended for chronic depression in TTad (i.e. no ‘step 2’), and instead, it is recommended that cognitive‐behavioural therapy should cover related maintenance processes including avoidance, rumination and interpersonal difficulties (NHS Futures, 2024). A focus on addressing unhelpful maintenance processes rather than targeting dysfunctional cognitive content is seen in various transdiagnostic, contextual, process‐focussed approaches to therapy. One example is Acceptance and Commitment Therapy (ACT), which has shown benefits in the carer literature (Collins & Kishita, 2019; Kishita et al., 2018; Losada et al., 2011; Sun, Ji, Leng, Li, et al., 2022), but is not endorsed in the current TTad manual (v7 NHS England, 2024).

Interpersonal difficulties may be considered with an individual or jointly with a partner who is willing to jointly engage in therapy. The recommended offers in TTad are behavioural couple therapy (BCT) or couple therapy for depression (CTD) (TTad Manual, v7, p. 23), irrespective of the non‐referred partner's outcome measure scores. The TTad manual also states that therapists should consider whether the non‐referred partner is also experiencing depression or anxiety and that data should be recorded for both partners with a separate record for each. Involvement of a care‐partner in therapy is common in psychological interventions in dementia, with family carers joining sessions either as co‐clients or to support the engagement of the person with dementia (e.g. acting as a memory‐aid or to facilitate communication). We did not find reviews with specific reference to either BCT or CTD protocols, although a scoping review of couple work in dementia identified 34 studies (Gilbert et al., 2023).

Symptoms of depression commonly co‐occur with anxiety in people affected by dementia (Romero‐Moreno et al., 2021). In TTad, there is a recommendation that clinicians who have identified depression in a service user should also assess for an underlying anxiety condition, as these are often under‐diagnosed. Co‐occurring symptoms of anxiety and depression are only labelled *co‐morbid* where diagnostic criteria are reached for both a depressive and anxiety disorder. The term ‘mixed anxiety and depression’ can be used where both are sub‐syndromal (NHS England, 2024). TTad services do not expect to see many individuals with [mixed anxiety and depression disorder] (NHS Futures, 2024) but, where it is identified, recommend Computerised CBT (cCBT; NHS England, 2024; NHS Futures, 2024). Importantly, *clinician support* is part of the TTad cCBT recommendation, in line with findings in this review and recent large trials in the UK (Fossey et al., 2021; Windle et al., 2025). Within the carer intervention literature, education about dementia and psychoeducation on carer stress are a typical components of cCBT alongside signposting to resources and relevant carer and dementia support services. Such elements are not standard TTad cCBT packages. TTad practitioners may require additional training and supervision beyond the dementia awareness training now mandatory for TTad staff (NHS England, 2024).

The interventions in the included reviews often differed in intensity to those currently delivered in TTad. By definition, people with dementia have significant decline in two or more areas of neurocognition (e.g. memory, perception, receptive and expressive language, cognitive speed and flexibility, and ‘executive’ functions such as planning, organisation and use of feedback). Necessarily, therefore, interventions in trials focussing on people with dementia include adaptations for neurocognitive impairments, either as standard elements of the protocol or tailoring to meet individual need. Such adaptations may include: a focus on behavioural rather than cognitive strategies (e.g. the ‘Peaceful Mind’ programme; Paukert et al., 2010, 2013), inclusion of neurocognitive‐enhancement skills (e.g. Charlesworth et al., 2015; Spector et al., 2015) or changing environmental contingencies (e.g. PATH; Kiosses et al., 2015; Howard et al., 2024). Whilst CBT‐informed approaches such as StaRt and PATH include techniques familiar to practitioners delivering LI interventions in TTad, protocols include more sessions than would be commonly be available for LI CBT (Cannon et al., 2024). In addition, home visits are often standard practice during research trials, whereas TTad services generally provide interventions through video conference, telephone or clinic visits. The TTad PPG recommends that services are commissioned in a way that allows home visits and that appropriate supervision should be in place for such visits (BABCP, 2024; NHS England, 2024). Incorporation of the evidence‐based protocols identified in this review would require an increase in the time typically allocated to LI workers to allow for travel to home visits, delivery of more sessions and time with an appropriately experienced clinical supervisor.

For people with dementia, no specific protocols are cited in the Older Adult TTad PPG, but recommendations include both ‘structured CBT with a focus on practical and pragmatic problem‐solving’ and ‘space for exploring personal experience of the diagnosis and associated losses’. People with dementia can be painfully aware of their current or future decline in abilities, and family carers commonly describe ‘losing’ the person that they once knew and the relationship that they had with them (Pozzebon et al., 2016). Carers can experience *anticipatory grief* whilst the person with dementia is still alive and then grieve again following their death. In the ‘additional considerations for depression’ section of the TTad Treatment Options guidance (NHS Futures, 2024), it is noted that recent bereavement may attract the problem descriptor ‘adjustment disorder’, or that loss may trigger depression and that in such circumstances, either PCECfD or IPT might be helpful alternatives to CBT‐based interventions (NHS Futures, 2024). We did not find reference to PCDCfD in our included meta‐analyses. IPT studies were included, but not separately meta‐analysed. For family caregivers, an initial RCT has indicated that a grief‐focussed, compassion‐based intervention reduced grief experience (Jahani et al., 2022).

A final challenge for bridging between the literature unpinning TTad and the psychological intervention literature for people affected by dementia is the choice of outcome measures. Whereas anxiety and depression are the primary outcomes in TTad, it is often measures of broader transdiagnostic constructs such as stress and burden that are used as primary outcomes in carer interventions, with quality of life being a key domain for people with dementia. Whilst the PHQ‐9 and GAD‐7 are fundamental to the TTad minimum dataset, they were all but absent from the psychological intervention literature for people with dementia or family carers. Measures used in treatment trials with people with dementia are often designed in a way that reduces cognitive load. For example ‘yes/no’ formats may be used rather than numbered scales, carers may act as proxies or observational tools may be employed. Such approaches were evident in our included reviews.

### Strengths and limitations

A strength of this review is the use of meta‐analyses of randomised controlled trials as our evidence base. However, the review is limited by the scoping nature of the searches. We have neither calculated overlap between reviews nor undertaken formal quality rating of the meta‐analyses (although we excluded those that did not compare intervention to control). The poor quality of psychosocial research in dementia and caregiving has been criticised previously, with numerous calls for improvement. Although there are some signs of improved methodology over time, much primary research continues to be under‐powered and poorly described. Furthermore, to increase the number of included studies, reviews of interventions for dementia also include studies of people with mild cognitive impairment (MCI) or people with dementia living in care homes. Although neurocognitive competences can be similar for people diagnosed with MCI and those in early stages of dementia, the psychological positioning of the two is different. Similarly, whilst people living at home or in Homes may have similar degrees of neurocognitive impairment and dependence on others, their environmental and social contexts are very different. We have been unable to draw any conclusions relating to ‘what works for whom’ as review outcomes were not considered in relation to participant characteristics (such as kinship, type of dementia, duration of caregiving, chronicity of depression, physical and mental health comorbidities).

### Implications

Considering psychological interventions in dementia through a TTad lens raises implications for clinical practice, policy and future research. There is an urgent need for: a carer identifier in the TTad minimum dataset to facilitate the generation of practice‐based evidence; dementia to be incorporated into the long‐term conditions (LTC) pathway; TTad guidance for a consistent approach to reducing assessment burden; and increased consideration of intersectionality and diversity.

The term ‘carer’ can be alienating where the person providing support does not self‐identify as a carer, as is often the case for spouses or other life partners, or where the individual has a strong kin‐identity in combination with a cultural or family heritage with strict expectations of family roles (Dowson et al., 2025). The term ‘carer’ can also mask the nature of the prediagnostic kinship. However, limitations of research evidence cannot be addressed through practice‐based evidence without a marker for carer status in the TTad minimum dataset, and the introduction of such a marker is long overdue.

Dementia is included in the Major Conditions Strategy (Department of Health and Social Care, 2023) alongside cardiovascular diseases, chronic respiratory diseases and musculoskeletal disorders. It has, however, not yet been included in the TTad LTC pathway or training curriculum (NHS England, 2018). The person‐centred, compassionate care described in the values statement of the LTC pathway implementation guidance aligns with the longstanding person‐centred approach in dementia care. Psychological practitioners working with people with dementia and family carers need to consider access, co‐occurring mental and physical health issues, and carer support, with implications for working transdiagnostically and for linking with other services and agencies—issues already encompassed within the TTad LTC pathway.

The TTad PPG for working with Older Adults (BABCP, 2024) recommends practitioners ‘make considerations that will allow those with dementia to engage with treatment’ (e.g. home visits, appointment reminders, session recaps, adjusting the pace, duration and frequency of sessions). However, although flexibility is recommended, there is no guidance on when an increased number of sessions might be justified or on how to address the known barrier to TTad presented by the weekly completion of minimum dataset questionnaires (Baker et al., 2022; Cheston & Howells, 2016). Enhancing the accessibility of TTad services has also been considered for other groups, for example people with intellectual disabilities, Veterans, Black, Asian and minority ethnic communities, with some suggestions that would also be relevant to people with dementia and family carers (e.g. additional sessions). There are well documented barriers to people from minoritised communities accessing dementia research, interventions and NHS services within the UK (e.g. Johl et al., 2016; Lillekroken et al., 2023; Waheed et al., 2020). Recommendations within the TTad PPG for Working with Black, Asian and Minority Ethnic Communities include local community engagement and outreach and ensuring more people from ethnic minority backgrounds are involved in all aspects of service development. Recommendations for adapting clinical practice include alignment with the beliefs, values and cultural and spiritual perspectives of minoritised communities, using interpreters where appropriate and providing staff training. Similar calls for culturally relevant community‐based interventions in relevant languages have been made for people affected by dementia (e.g. Kenning et al., 2017; Parveen et al., 2017), and there are existing examples of carer interventions being adapted to increase cultural relevance (e.g. Au et al., 2010; Dowson et al., 2025; Webster et al., 2023). Working with community and faith groups and offering interventions with a less explicit emphasis on mental health (and more on supporting ‘good care’, communication and good relationships, etc) could reduce barriers.

Underserved and minoritised individuals are not limited to ethnically minority communities (Witham et al., 2020), and intersecting identities can mean some individuals experience multiple challenges in accessing services. Further attention should be paid to diversity within people affected by dementia and the potential for interventions attuned to specific individual characteristics or circumstances. Not specifically mentioned in the TTad PPGs and under‐researched in the context of carers and people living with dementia, it is also important to consider those who identify as LGBTQ+. Evidence suggests that for LGBTQ+ older adults, individuals can face discrimination in familial relationships (Averett et al., 2011), may not always disclose sexual and/or gender identity as a form of self‐protection and face barriers to service access (Chen et al., 2022; Smith et al., 2024), with LGBTQ+ ethnically minoritised individuals sometimes experiencing additional barriers to service access and wellbeing (Chen et al., 2022). However, more research and consideration are needed to understand these diverse and intersectional identities and generate specific recommendations (Smith et al., 2024).

## CONCLUSIONS AND FUTURE DIRECTIONS

Much has changed since the last update of NICE guidance for dementia (NG97; National Institute for Health and Care Excellence, 2018). A revised NICE guideline should update the evidence base for psychological interventions for people with dementia and family carers and be written in a format that can be adopted by TTad. When the PPG documents are revised, guidance for working with people with progressive cognitive impairments should potentially be separated from the guidance for older adults, as not all people with dementia are ‘older’, and including both in the guideline can lead to false assumptions about younger people with dementia and older people without dementia.

Future directions for research should include approaches for addressing anxiety, especially for people with dementia. Similar to the findings from this review, a recent meta‐analytic review specifically on interventions for anxiety in dementia suggests that the answer may lie outside talking therapies, with promising approaches including music therapy, muscular approaches or stimulating cognitive or physical activities (Nimmons et al., 2024). For both people with dementia and carers, interest is increasing in compassion‐based work (Collins et al., 2018; Madani & Farokhzad, 2025; Spector et al., 2024; Zhu et al., 2024) and new protocols being developed for mindfulness (e.g. Sánchez‐Pérez et al., 2024) and ACT (e.g. Atefi et al., 2025). Interventions to enhance mentalising and communication also show promise, for example the multi‐component group‐based transdiagnostic intervention ‘Empowered Conversations’ that includes responses to specific interpersonal, communication and grief issues that can arise for carers (Morris et al., 2020).

In conclusion, to improve access to TTad for people with dementia and family carers, changes are required to TTad data collection and service delivery and to research evaluations with people affected by dementia. However, whilst it is important that psychological therapies should be available to people affected by dementia, we cannot expect psychological therapy alone to meet the wide‐ranging needs of this complex population. Ultimately, offering community‐led options and reducing barriers to existing statutory services are both needed.

## AUTHOR CONTRIBUTIONS

**Georgina Charlesworth:** Conceptualization; methodology; writing – original draft; writing – review and editing; formal analysis. **Cassie Eastham:** Writing – original draft. **Lydia Morris:** Conceptualization; methodology; writing – original draft; writing – review and editing; formal analysis; supervision.

## CONFLICT OF INTEREST STATEMENT

The authors declare no conflicts of interest.

## Data Availability

Data sharing not applicable to this article as no datasets were generated or analysed during the current study.
